# Changes in health-related quality of life are associated with patient satisfaction following total hip replacement: an analysis of 69,083 patients in the Swedish Hip Arthroplasty Register

**DOI:** 10.1080/17453674.2019.1685284

**Published:** 2019-11-04

**Authors:** Gabrielle S Ray, Philip Ekelund, Szilard Nemes, Ola Rolfson, Maziar Mohaddes

**Affiliations:** a Harris Orthopaedic Laboratory, Massachusetts General Hospital, Boston, USA;; b Sahlgrenska University Hospital, Department of Orthopaedics, Gothenburg, Sweden;; c Swedish Hip Arthroplasty Register, Gothenburg, Sweden;; d Department of Orthopaedics, Institute of Clinical Sciences, The Sahlgrenska Academy, University of Gothenburg, Gothenburg, Sweden

## Abstract

Background and purpose — Total hip replacement (THR) aims mainly to improve quality of life via restoration of hip function and provision of pain relief. This study sought to assess whether improvements in quality of life between the preoperative and 1-year postoperative period were associated with patient satisfaction

Patients and methods — Data were extracted for 69,083 THR operations with complete data reported to the Swedish Hip Arthroplasty Register (SHAR) between 2008 and 2015. Health-related quality of life and patient satisfaction were captured using the Euro-Qol-5D (EQ-5D) and visual analogue scale (VAS), respectively. Multivariable analysis was performed to assess associations between the changes in pre- and postoperative EQ5D and patient satisfaction.

Results — In patients reporting severe or moderate problems with mobility preoperatively, improvement to no problems was associated with numerically higher patient satisfaction (coefficient –18 [95% CI –22 to –14] and –18 [–18 to –17]). Improvement in the self-care dimension from severe or moderate problems to no problems was associated with numerically higher patient satisfaction (–15 [–16 to –14] and –13 [–15 to –11]). Improvement from severe problems with the ability to perform usual activities to no problems was associated with numerically higher patient satisfaction (–18 [–19 to –17]). This association was also found for improvement in pain/discomfort and anxiety/depression (–16 [–17 to –15] and –15 [–16 to –14]).

Interpretation — Our results indicate that satisfaction with the operated hip is a valid patient-reported outcome reflecting the changes in different EQ-5D dimensions and should be included in the follow-up of patients after THR surgery.

The 2 main goals of total hip replacement (THR) are pain relief and restoration of hip function (Pivec et al. 2012). The success of total joint replacement has largely been measured through implant survivals and the 10-year success rate of THR has been reported to be as high as 95% (Pivec et al. 2012). Yet, 10–15% of patients undergoing THR report persistent pain and functional limitation postoperatively (Nikolajsen et al. 2006). It could be argued that using only implant survival analysis fails to identify patients who achieve the 2 primary goals of surgical intervention.

These shortcomings of implant survival analysis have been identified (Wylde and Blolm 2011) and patient-reported outcome measures (PROMs) have been used as a more adequate indicator of surgical success from the patient’s perspective. PROMs and patient satisfaction measures are increasingly being utilized by surgeons, hospitals, insurance companies, and health policy-makers to monitor quality of services (Baumann et al. 2009). Patient satisfaction is known to be a combination of subjective and social-cultural feelings with various cognitive, behavioral, and psychological influences (Brokelman et al. 2012). Thus, it is critical to understand what this metric truly captures in patients following THR surgery with respect to hip function and pain relief (Baumann et al. 2009).

Since 2002, the Swedish Hip Arthroplasty Register (SHAR) has instituted a standardized PROMs program in the follow-up of all THR patients. A visual analogue scale (VAS) addressing patient satisfaction with the outcome of the surgical intervention is obtained in the postoperative PROMs questionnaire. The VAS satisfaction is a simple instrument that has demonstrated good validity and reliability (Brokelman et al. 2012). It is unknown how accurately this measure reflects the outcome of the surgery with respect to pain relief and restoration of hip function.

The SHAR captures health-related quality of life using the Euro-Qol-5D (EQ-5D), which assesses the patient’s current health in 5 dimensions: mobility, self-care, usual activities, pain/discomfort, and anxiety/depression. Patients grade their current level of function in each dimension into 1 of 3 levels of disability (none, moderate, or severe) (EuroQolGroup 1990).

This study sought to assess if changes observed in the 5 dimensions of the EQ-5D between the preoperative and postoperative period were associated with patient satisfaction 1 year following THR.

## Patients and methods

The Swedish Hip Arthroplasty Register collects data on all patients undergoing THR in Sweden including date of birth and sex, diagnoses, type of implant and fixation method used, ASA classification, height, and weight. Additionally, since 2002 PROMs are administered to all patients preoperatively and, unless revised, at 1, 6, and 10 years postoperatively. These PROMs consist of a 10-item questionnaire including Charnley’s functional categories (A, B, and C) (Callaghan et al. 1990), VAS for pain, and the EQ-5D instrument. In the postoperative intervals, a VAS for satisfaction is also collected, which ranges from 0 (satisfied) to 100 (dissatisfied). The vertical line is supplemented by subscale indicators for ordered response levels (0 to 20, very satisfied; 20–40, satisfied; 40–60, uncertain; 60–80, not satisfied; and 80–100, dissatisfied) (Rolfson et al. 2011).

**Figure 1.**
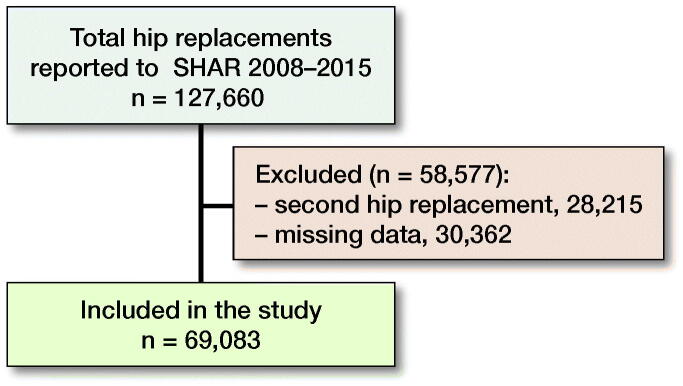
Flowchart of patients receiving THR enrolled in the Swedish Hip Arthroplasty Register between 2008 and 2015 meeting inclusion criteria for the present study.

Data were extracted for all 127,660 THR surgeries reported to SHAR between 2008 and 2015 (Figure 1). In patients having both hips operated during the study period only the first operated hip was included. 69,083 cases were included in the present study (Table 1).

**Table 1.** Demographics of patient population included in the study

Variable	
Age, mean (SD)	68 (10)
Women n (%)	38,994 (56)
Men, n (%)	30,089 (44)
Height (cm), mean (SD)	170 (10)
Weight (kg), mean (SD)	79 (16)

### Statistics

For each EQ-5D dimension, data were recorded for the 3 levels of disability (1= none, 2 = moderate, and 3 = severe). Changes between the preoperative and postoperative EQ-5D were then calculated. For each patient, responses to each EQ-5D component were directly compared between preoperative and postoperative questionnaires and improvement; stagnation or decline were presented as proportions. Multivariable regression models were used to examine relationships between the satisfaction VAS obtained 1 year postoperatively and the categorical changes in each EQ-5D dimension. 5 different models were thus built, one for each EQ-5D dimension. Satisfaction VAS was used as a continuous outcome variable. Age and sex were controlled for in all 5 regression analyses. Patients reporting moderate problems both preoperatively and 1 year postoperatively (i.e., 2 to 2) were used as the reference group. Unstandardized regression coefficients were obtained for each categorical change in EQ-5D components (e.g., moderate problems to no problems). This means these coefficients are measured in the same units as the outcome. Thus, the regression coefficients should be interpreted as the adjusted deviation from the reference value. The reference value was always the VAS satisfaction of patients who reported moderate problems in that respective EQ-5D domain both prior and after the surgery. The VAS Satisfaction ranges between 0 and 100, with 0 representing the best possible outcome and 100 the worst possible outcome. Statistical analyses were conducted using R version 3.4.2 (R Foundation for Statistical Computing, Vienna, Austria).

### Ethics, funding, and potential conflicts of interest

As this was a prospective observational register study, no additional intervention was necessary. Each patient voluntarily participated in the questionnaire and all personal data are aggregated to ensure patient de-identification. Patients also have the right to leave the PROM-program at any time. The study is part of a large research project, which has been approved by the Regional Ethical Review Board in Gothenburg (entry number 271-14). There was no funding for this project and no conflicts of interest.

## Results

### EQ-5D differences

The majority of patients reported moderate or severe problems preoperatively in the mobility, usual activities, and pain/discomfort dimensions of the EQ-5D. Problems with self-care or anxiety/depression preoperatively were less common (Table 2). The greatest improvement 1 year postoperatively was seen in the pain/discomfort dimension as 66% of patients reported improvement, 32% reported no change, and 2% reported worse problems with pain/discomfort. 55% of patients reported improvements in mobility, 19% reported improvements with respect to self-care, 47% reported improvements in the ability to perform usual activities, and 28% reported improvements in the anxiety/depression dimension.

**Table 2.** Distribution of reported problems with respect to all 5 EQ-5D dimensions preoperatively and 1-year following THR

	Preoperative EQ-5D			1-year Postoperative EQ-5D		
Factor	No problem	Moderate problems	Severe problems	No problems	Moderate problems	Severe problems
Mobility	5,250 (8)	63,557 (92)	276 (0.4)	41,863 (61)	27,116 (39)	104 (0.2)
Self-care	52,874 (77)	15,562 (23)	647 (0.9)	63,811 (92)	4,865 (7.0)	407 (0.6)
Usual activity	26,335 (38)	35,414 (51)	7,334 (11)	53,480 (77)	14,145 (21)	1,458 (2.1)
Pain/discomfort	1,042 (1.5)	39,205 (57)	28,836 (42)	30,357 (44	35,440 (51)	3,286 (4.8)
Anxiety/depression	39,604 (57)	27,032 (39)	2,447 (3.5)	53,978 (78)	14,020 (20)	1,085 (1.6)

### Associations between changes in EQ-5D and VAS satisfaction

In patients reporting problems with mobility preoperatively, improvement to no problems with mobility at 1 year postoperatively was associated with higher patient satisfaction (Table 3, see Supplementary data). Patients who reported severe problems with mobility pre- and postoperatively had greater dissatisfaction with their THR procedure and patients reporting deterioration from no problems to severe problems with mobility had the greatest dissatisfaction with their THR procedure (Figure 2A).

Figure 2.Forest plot of the multivariable analysis of the association between patient satisfaction and changes in the pre- and postoperative EQ-5D: (A) mobility dimension; (B) self-care dimension; (C) usual activities dimension; (D) pain/discomfort dimension; and (E) anxiety/depression dimension. Coefficient values are given on the right-hand side of the figure.

**Figure:**
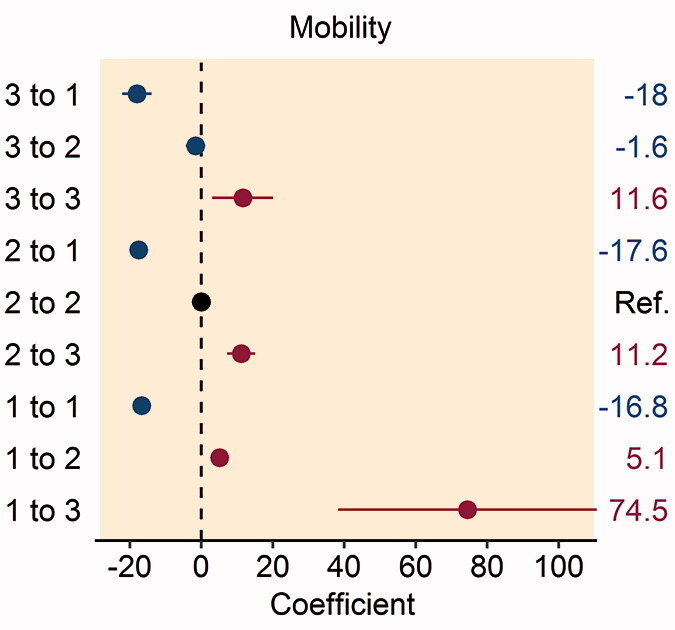

**Figure:**
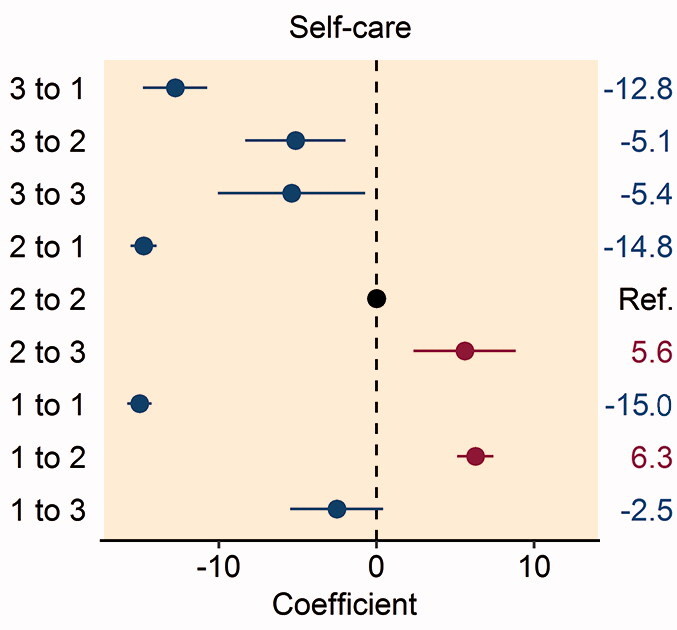

**Figure:**
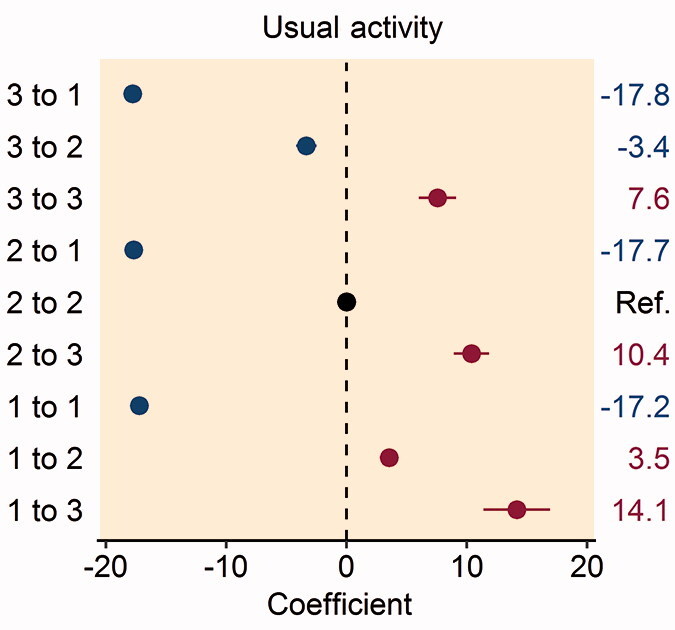

**Figure:**
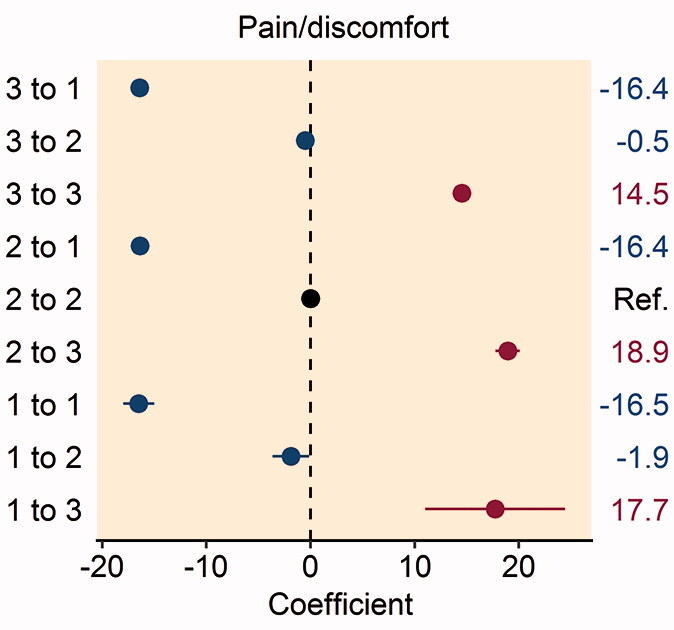

**Figure:**
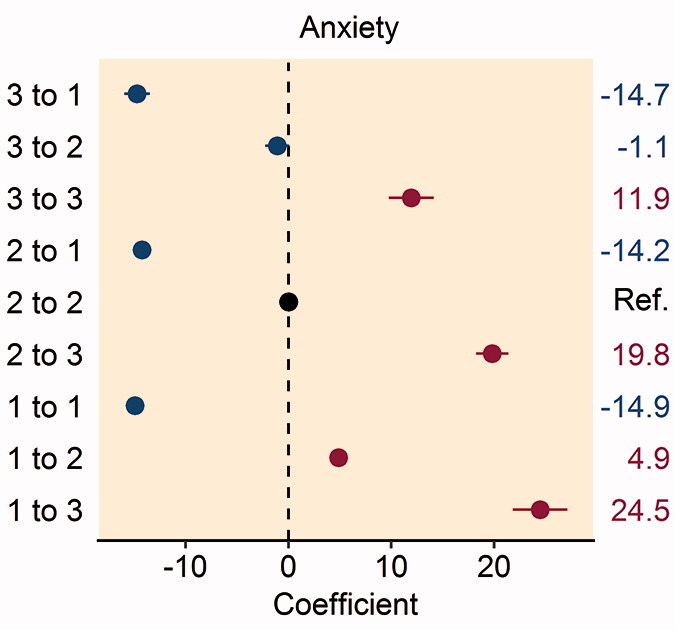

Improvement in the self-care dimension from moderate or severe problems preoperatively to no problems 1 year following THR was associated with higher patient satisfaction (Table 4, see Supplementary data). Patients who worsened between the preoperative period and 1 year following their THR from no problems to moderate problems or moderate problems to severe problems with self-care had greater dissatisfaction with their THR procedure (Figure 2B).

Patients reporting any improvement in their ability to perform usual activities 1-year following THR had greater patient satisfaction with their procedure, with the highest patient satisfaction found amongst those who reported severe problems in this domain preoperatively and improved to no problems postoperatively (Table 5, see Supplementary data). Conversely, any patient reporting worsening difficulty with their ability to perform usual activities had greater dissatisfaction with their THR procedure, particularly in patients worsening from no problems to severe problems postoperatively (Figure 2C).

Patients reporting moderate or severe pain/discomfort preoperatively with improvement to no pain postoperatively had greater satisfaction with their THR procedure (Table 6, see Supplementary data). Patients reporting worsening pain/discomfort from no problems to severe problems, or moderate problems to severe problems postoperatively had greater dissatisfaction following their THR procedure (Figure 2D).

In patients reporting moderate or severe anxiety/depression preoperatively, improvement to no problems 1 year postoperatively was associated with higher patient satisfaction (Table 7, see Supplementary data). Patients reporting any worsening of anxiety/depression had greater dissatisfaction following their THR procedure, particularly patients who reported no problems preoperatively and severe problems postoperatively (Figure 2E). 

## Discussion

Patient satisfaction is an important metric now collected in the follow-up of arthroplasty procedures to assess subjective outcomes. The satisfaction VAS is a simple instrument used to quantify patient satisfaction after THR. This score has demonstrated good validity and reliability (Brokelman et al. 2012). In the Swedish Hip Arthroplasty Register, the satisfaction VAS, as well as the EQ-5D, is collected postoperatively to measure surgical success from the patient’s perspective (Rolfson et al. 2011). It is currently unknown how well patient satisfaction mirrors changes reported in the EQ-5D. Our study sought to investigate the relationship between the satisfaction VAS obtained 1 year postoperatively and the changes between the pre- and postoperative scores in the different EQ-5D dimensions. We hypothesized that there would be high correlations between the satisfaction VAS and the degree of improvement in health-related quality of life.

Our study indicated strong relationships between the satisfaction VAS and changes in each dimension of the EQ-5D. For all 5 dimensions, patient satisfaction was associated with improvement from severe problems preoperatively to no problems 1 year postoperatively. Conversely, patient dissatisfaction was associated with deterioration from no problems to severe problems in 4 of the 5 dimensions: usual activity, pain/discomfort, mobility, and anxiety/depression. For all 5 dimensions, patients reporting no problems postoperatively reported high satisfaction with the most recent treatment of their hip. Regardless of preoperative scores, patients reporting severe problems in usual activities, pain/discomfort, mobility, and the anxiety/depression dimensions 1 year after THR more frequently reported dissatisfaction with their surgical intervention.

The SHAR appropriately measures the quality of surgical intervention by assessing restoration of hip function and pain relief through the EQ-5D instrument, a VAS for pain, and a VAS for satisfaction. Patient satisfaction has proven to be a multi-faceted expression of affective, cognitive, and subjective feelings (Brokelman et al. 2012). Previous literature has demonstrated that the patient’s mental well-being and preoperative expectations contribute to their satisfaction with their surgical intervention (Bourne et al. 2010). Preoperative anxiety has been associated with lower rates of patient satisfaction following THR (Rolfson 2010). Our study similarly identified that patients with persistent or increased anxiety between the pre- and postoperative periods have less satisfaction following THR. However, patients with preoperative anxiety, either moderate or severe, who report no anxiety 1 year postoperatively have increased patient satisfaction.

A high proportion of patients reported moderate or severe problems with anxiety/depression preoperatively (n = 29,479, 43%). This could be due to the fact that 99% of patients were reporting moderate or severe pain and 92% reported limited mobility, signifying that there was substantial impairment in quality of life, or indicative of the high degree of anxiety that could accompany the natural stress all patients endure prior to having a surgical procedure. This may also be due to the inherent limitation of the EQ-5D 3-level questionnaire. The response options for patients are “I am not anxious or depressed,” “I am moderately anxious or depressed,” or “I am extremely anxious or depressed.” These 3 options fail to distinguish between moderate anxiety prior to a surgical intervention and persistent moderate anxiety and depression.

To our knowledge, no studies have previously assessed how the satisfaction VAS reflects the changes in the EQ-5D between the preoperative and postoperative time periods in patients undergoing THR. In a study of patients undergoing total knee replacement surgery, patient dissatisfaction was found to be related to lack of improvement in preoperative status in pain scores and functional scores; however, patient satisfaction was assessed using a questionnaire allowing patients to only respond “Yes”, “No,” or “I’m not sure” (Jacobs and Christensen 2014). This limits its extension to the present study.

Prior literature has identified age and sex as predictive variables for patient satisfaction following THR (Palazzo et al. 2014, Schaal et al. 2016). Thus, we included age and sex in all regression analyses to account for their confounding nature. Additional studies have demonstrated that symptomatic arthritis in another large joint is predictive of dissatisfaction with THR at 1 year postoperatively (Anakwe et al. 2011). Unfortunately, we could not account for that as the data were extracted from the SHAR, which does not have this detailed clinical information for all patients. This, and identification of other variables associated with patient satisfaction after THR, should be the focus of future research. Additionally, future research should investigate whether other regularly utilized PROMs, such as the Harris Hip Score, are associated with quality of life.

Our study has some limitations. First, the EQ-5D 3 level questionnaire might not be an optimal instrument to discern minor disabilities. For example, with respect to the pain dimension, if a patient has only minor pain occasionally, neither the option of “no pain” nor that of “moderate pain” truly captures the patient’s experience. A questionnaire with more alternatives for each dimension might be more effective for capturing all levels of disability; however, the time and effort required to fill in such a form may lower response rates. Second, as the EQ-5D is a generic instrument designed to capture health-related quality of life, it is difficult to determine patient responses that are strictly attributable to their hip disease. For instance, in our study some patients reported increased pain following THR surgery and it is possible that the increased pain reported is related to another diseased joint. Additionally, like most registry studies, our study suffers from some loss to follow-up. Of the 99,445 eligible patients with unilateral hip replacement, only 69,083 patients had complete PROMs for analysis. The 30,362 patients excluded with missing PROMs may have had higher or lower baseline QoL characteristics than the studied population, therefore this may bias the results. Finally, as the patients we included had hip replacements between 2008 and 2015, it is possible that patient expectations changed during that time period affecting quality of life and satisfaction; however, we believe that the robust sample size generated from this study period strengthens the study and arthroplasty practices did not change drastically in this time period.

Our study draws on a large, nationwide register that is a reliable method for data capture and is the largest study conducted on this topic to date. As modern medicine relies increasingly on patient-reported outcomes as a tool to gauge success and quality of care, it remains paramount to better understand factors predictive of patient satisfaction. We were able to demonstrate clear associations between satisfaction VAS and changes in the different dimensions of the EQ-5D. Our results indicate that satisfaction with the operated hip is a valid patient-reported outcome reflecting the changes in different EQ-5D dimensions and should be included in the follow-up of patients following THR surgery.

### Supplementary data

Tables 3–7 are available as supplementary data in the online version of this article, http://dx.doi.org/10.1080/17453674.2019. 1685284.

## Supplementary Material

Supplemental Material
