# Supplementary material for: Changes in health-related quality of life are associated with patient satisfaction following total hip replacement: an analysis of 69,083 patients in the Swedish Hip Arthroplasty Register
Source: Acta Orthop. 2019 Nov 4;91(1):48–52. doi: 10.1080/17453674.2019.1685284 (PMC7008235; doi:10.1080/17453674.2019.1685284)
Supplement: Supplemental Material [file IORT_A_1685284_SM3712.pdf]

Supplementary data

Table 3. Multivariable analysis of the association between patient satisfaction and changes in the pre- and postoperative EQ-5D mobility dimension, controlling for age and sex

Preoperative mobility status<br>Postoperative status	Coefficient (95% CI)
No problems	
No problems	–17 (–17 to –16)
Moderate problems	5 (4 to 6)
Severe problems	75 (38 to 111)
Moderate problems	
No problems	–18 (–18 to –17)
Moderate problems [a]	– –
Severe problems	11 (7 to 15)
Severe problems	
No problems	–18 (–22 to –14)
Moderate problems	–2 (–4 to 1)
Severe problems	12 (3 to 20)

[a] Reference value

Table 4. Multivariable analysis of the association between patient satisfaction and changes in the pre- and postoperative EQ-5D self-care dimension, controlling for age and sex

Preoperative self-care status<br>Postoperative status	Coefficient (95% CI)
No problems	
No problems	–15 (–16 to –14)
Moderate problems	6 (5 to 7)
Severe problems	–3 (–6 to 0.4)
Moderate problems	
No problems	–15 (–16 to –14)
Moderate problems [a]	– –
Severe problems	6 (2 to 9)
Severe problems	
No problems	–13 (–15 to –11)
Moderate problems	–5 (–8 to –2)
Severe problems	–5 (–10 to –1)

[a] See Table 3

Table 5. Multivariable analysis of the association between patient satisfaction and changes in the pre- and postoperative EQ-5D usual activities dimension, controlling for age and sex

Preoperative usual activities status<br>Postoperative status	Coefficient (95% CI)
No problems	
No problems	–17 (–18 to –17)
Moderate problems	4 (3 to 4)
Severe problems	14 (11 to 17)
Moderate problems	
No problems	–18 (–18 to –17)
Moderate problems [a]	– –
Severe problems	10 (9 to 12)
Severe problems	
No problems	–18 (–19 to –17)
Moderate problems	–3 (–4 to –3)
Severe problems	8 (6 to 9)

[a] See Table 3

Table 6. Multivariable analysis of the association between patient satisfaction and changes in the pre- and postoperative EQ-5D pain/discomfort dimension, controlling for age and sex

Preoperative pain/ discomfort status<br>Postoperative status	Coefficient (95% CI)
No problems	
No problems	–17 (–18 to –15)
Moderate problems	–2 (–4 to 0)
Severe problems	18 (11 to 25)
Moderate problems	
No problems	–16 (–17 to –16)
Moderate problems*	– –
Severe problems	19 (18 to 20)
Severe problems	
No problems	–16 (–17 to –16)
Moderate problems	–1 (–1 to 0)
Severe problems	15 (14 to 15)

[a] See Table 3

Table 7. Multivariable analysis of the association between patient satisfaction and changes in the pre- and postoperative EQ-5D anxiety/depression dimension, controlling for age and sex

Preoperative anxiety/ depression status<br>Postoperative status	Coefficient (95% CI)
No problems	
No problems	–15 (–15 to –15)
Moderate problems	5 (4 to 6)
Severe problems	25 (22 to 27)
Moderate problems	
No problems	–14 (–15 to –14)
Moderate problems*	– –
Severe problems	20 (18 to 21)
Severe problems	
No problems	–15 (–16 to –14)
Moderate problems	–1 (–2 to 0)
Severe problems	12 (10 to 14)

[a] See Table 3
